# 

**Published:** 1895-02

**Authors:** 


### CONTENTS.

ORIGINAL ARTICLES:

Horseflesh for Our Food. By RUSH SHIPPEN HUIDEKOPER, M.D. . . . 65
Meat Inspection. By CLAUDE D. MORRIS, V.S. . . . . . . . . 71
Remarks on Southern Cattle Fever. By W. H. HARBAUGH, V.S. . . . 78
Poisoning of Cattle with Nitrate of Sodium. By JAS. B. PAIGE, D.V.S. . . 89
Canine Distemper: Its Causes, Symptoms, Sequels, and Treatment. By E. WADAMS, V.S. . . . . . . . . . . . . . . . 92
Notes on Tuberculosis. By E. P. NILES, D.V.M. . . . . . . . 99
Operation for Radical Cure of Umbilical Hernia in a Colt Ten Months Old. By A. W. CLEMENT, V.S. . . . . . . . . . . . . . . 104

SELECTIONS:

State Legislation—New York . . . . . . . . . . . . . 106

REPORTS OF CASES:

Treatment for Azoturia. By LOUIS OLRY LUSSON . . . . . . . 110
An Interesting Case. By LOUIS OLRY LUSSON . . . . . . . . 110

EDITORIAL:

Veterinary Legislation in New York—New York Desires a Higher Standard—Necessity for a Uniform Degree or Title—Error . . . . . . . . 112

REVIEWS:

A Manual of Veterinary Therapeutics . . . . . . . . . . 113
Manual of Veterinary Micro-Biology . . . . . . . . . . 114

REVIEW OF VETERINARY SANITARY WORK:

Infectiousness of Milk . . . . . . . . . . . . . 114
The Tuberculosis Commission . . . . . . . . . . . . 115

CORRESPONDENCE . . . . . . . . . . . . . . . . 116

SOCIETY PROCEEDINGS:

New York State Veterinary Medical Society . . . . . . . . 118
Massachusetts Veterinary Association . . . . . . . . . . 125
Virginia State Veterinary Medical Association . . . . . . . 127
Maine Veterinary Medical Association . . . . . . . . . . 128
Maryland Veterinary Medical Society . . . . . . . . . . 130
Michigan State Veterinary Medical Association . . . . . . . 131

